# An Essay on the Physiology, Hygiene, and Medical Management of the Epochs of Puberty and Cessation of the Menses in Women; and on the Periodical Discharge of Ova in the Human Female and in Mammifera

**Published:** 1845-01

**Authors:** 


### ART. IX.

1. *De la Puberté et de l'Age Critique chez la Femme, au point de vue physiologique, hygiènique, et médical, et de la Ponte périodique chez la Femme et les Mammifères.* Par M. A. RACIBORSKI.—*Paris*, 1844.

*An Essay on the Physiology, Hygiene, and Medical Management of the Epochs of Puberty and Cessation of the Menses in Women; and on the Periodical Discharge of Ova in the Human Female and in Mammifera.* By M. A. RACIBORSKI.—*Paris*, 1844. 12mo, pp. 534.

2. *Beweis der von der Begattung unabhängigen periodischen Reifung und Loslösung der Eier der Säugethiere und des Menschen als der ersten Bedeingung ihrer Fortpflanzung.* Von TH. S. W. BISCHOFF. —*Giessen*, 1844.

*Proof that the Periodical Maturation and Discharge of the Ova, independently of Coitus, in Mammalia and the Human Female, constitute the essential condition of their Propagation.* By Prof. BISCHOFF.—*Giessen*, 1844. 4to, pp. 54.

THE work of M. Raciborski consists of a reprint of several papers which appeared in one of the French medical journals in the autumn of last year, and forms the first part of a treatise on Menstruation and its Diseases, which he purposes completing speedily. Its most important parts are those which illustrate the periodical maturation and discharge of ova during menstruation in the human subject, or at the rutting season in animals; and in which the author endeavours to establish the close analogy between these two phenomena in the purposes which they subserve as well as in the occurrences by which they are attended.

The menstrual function has been for ages a theme on which physiologists have allowed their fancy to run riot; and the superstitions of the vulgar concerning it have often been but little more absurd than the hypotheses of the learned. Some have denied that it is a natural phenomenon at all, and have regarded it as one of the evil results of civilization; others have professed to see in it a part of the penalty inflicted on our first parent for her sin; while again others have pronounced it to be a beneficial arrangement for abating the ardour of the sexual feelings; and believe that it thus answers a high moral purpose. It has been said to be the means of getting rid of that superfluous blood which is employed during pregnancy in the nutrition of the fetus, and thus preventing plethora; while the writers of the chemical school propounded the theory, that it is a great means of effecting a monthly purification of the blood from all effete and noxious ingredients,—a hypothesis which tallies closely with the opinions of the vulgar. In the midst of these theories, and fifty others which it were bootless to detail, the points of resemblance between the menstrual function and the rut of animals did not escape notice. There were many points of dissimilarity, however, which, so long as the mere discharge of a certain quantity of blood was regarded as constituting the essence of menstruation, prevented the resemblance between the two functions being fully recognized, and the causes and signification of menstruation being understood.

It would occupy more space than we can allot to it, to trace ever so briefly the successive steps by which, setting out from little more than pure hypothesis, we have now arrived at almost demonstrative proof that "all the phenomena of menstruation depend upon the ovaria, and that at each period a Graafian vesicle bursts, and its contents escape." Many claimants participate in the honour of this discovery; but none except Professor Bischoff has done so much as M. Raciborski towards establishing satisfactorily the analogy between menstruation and the rut of animals, and proving that the phenomena attending both are but the outward expressions of those changes in the ovaria by which the periodical maturation and discharge of ova are effected. This subject occupies the latter part of M. Raciborski's book; in the first part he treats of the period of puberty, the management it requires, and the various circumstances which interfere with its occurrence. Many very unfounded notions have prevailed as to the period of puberty in different countries; it has been asserted to be extremely precocious in hot countries, and proportionately tardy in cold climates. Several of these opinions have been refuted by Mr. Roberton of Manchester; and the correctness of his views is in great measure confirmed by M. Raciborski. He conceives that the importance of all those causes which have been supposed to influence the period of puberty has been greatly exaggerated. There are two, however, which seem really to exert considerable influence on the maturity of the human female: the one, the temperature of the country; the other, the natural constitution of each individual. With reference to the former his very elaborate researches show that a difference of one degree in latitude usually gives rise to a difference of one month in the date of the first appearance of the menses; a fact very well shown in the following table:

| " Name of Town. | Latitude. | Age at first menstruation. | Mean annual temperature. |
|---|---|---|---|
| Toulon | 43 | 14·081 | 15· |
| Marseilles | 43 | 14·015 | 15· |
| Lyons | 46 | 14·492 | 11·6 |
| Paris | 49 | 14·465 | 10·6 |
| Göttingen | 52 | 16·038 | 8· |
| Warsaw | 52 | 15·083 | 9·2 |
| Manchester | 53 | 15·191 | 9·6 |
| Skeen | 59 | 15·450 | 6· |
| Stockholm | 59 | 15·590 | 5·7 |
| Sweedish Lapland | 65 | 18· | 4· " (p. 17.) |

From this table too we see that the difference is not produced by mere difference of latitude, but that it is rather to be attributed to the difference of temperature which is usually associated with it. Thus, for instance, menstruation takes place earlier at Warsaw than at Göttingen, though the latitude of the two places is the same; and earlier too at Manchester, although situated one degree further north than Göttingen. The influence of a high temperature in favouring the greater activity of the sexual functions is seen as well in the greater prolificness of the female as in the earlier occurrence of menstruation. M. B. de Chateauneuf, in a passage quoted by M. Raciborski, observes that,

"If we draw a line dividing Europe into two climates, one of which beginning at Portugal and ending at the Low Countries, would extend from 40 to 50°, and represent the south; while the other reaching from Brussels to Stockholm, or from 50 to 67°, would represent the north, we shall find that in the former, a hundred marriages yield 4·573 births to each marriage: while in the latter, the same number of marriages will yield only 4·3.

"This difference is still more striking if we compare the extreme temperatures together. Thus in Portugal there are born 5·10 children per marriage, in Sweden only 3·62." (p. 24.)

Individual differences, however, appear to be quite as influential as any general cause, which indeed they not unfrequently counteract. They are observed in all countries, under all varieties of latitude, in all classes of society in cities as well as in the country. "Thus at Skeen, in the neighborhood of Christiania, the average period of puberty is fifteen and a half years; but nevertheless" says M. Raciborski, "I met with four women in the hundred who had begun to menstruate at eleven." (p. 7.)

He carefully examines the influence of race in modifying the period of puberty, and shows that after the lapse of so many centuries, the Jews in Warsaw still retain somewhat of their oriental character, menstruation beginning a few months earlier, and likewise ceasing somewhat sooner than among the Christian inhabitants of the same city. The greater simplicity of a country life is followed by a retardation of the period of puberty, and M. Raciborski ascertained that while, in Paris, menstruation begins at 14·465 years, the age at which it occurs in the villages in the immediate neighbourhood of the capital is 15·020, showing a difference of nearly a year, for which no other cause can be assigned than the different mode of life.

He next notices (p. 77) cases of extraordinarily precocious menstruation of which he has collected many cases from various authors, and finds in the theory he has adopted with reference to the cause of menstruation, a ready explanation of these exceptional occurrences. Menstruation which is the index of aptitude for generation appears last in the train of changes, and when all the organs have acquired their due development. In some countries these changes take place earlier than in others, and there seems to be no reason for regarding menstruation during infancy as anything else than a highly exaggerated degree of precocious puberty.

In both cases the premature appearance of the menses seems to depend on the precocious development of the ova and of the Graafian follicles, which give, as we know, the impulse to all the other organs of the reproductive apparatus; only in the one case this precocity evidently depends on the influence of climate or temperature, while in the other it appears to be the effect of congenital peculiarities. One circumstance in favour of this opinion is the invariable coincidence of development of the mammæ and external genital organs with the hemorrhage, in cases of premature menstruation. These phenomena, indeed, do not occur until the follicles of Graaf are already on the point of attaining that full degree of development which characterizes the period of puberty; they are absent, or exist only in a rudimentary degree, whenever the development of these follicles is impeded by some primitive disposition of the economy; by diseases, or by the congenital absence of the ovaries, or their removal.

In support of this opinion too, he observes that though the Graafian follicles are usually not formed until after birth, he yet found a perfectly developed Graafian vesicle on one occasion in the ovary of a child still-born at the seventh month of pregnancy.

"Now everything is in favour of the supposition that had this child continued to live, it would have presented several follicles at the conclusion of pregnancy, and it is very probable that if the same degree of vital energy had continued, it would soon have arrived at the end of some months of extra-uterine life; at that state which is not usually attained before the age of fourteen or fifteen years. That which we observed as an exception to the general rule in the above-mentioned fact, may occur in all cases of premature menstruation. It may be said, indeed, that in all cases of this kind, the most important anomaly consists in the premature development and precocious maturity of the ova; the menstrual hemorrhage is only the necessary consequence of this anatomical and physiological peculiarity." (p. 68.)

It must not, however, be inferred that the converse of this holds good, and that in women in whom the occurrence of menstruation does not take place till a very late period, there is a corresponding delay in the maturity of the ova and Graafian follicles.

"So far, indeed, from that being the case, these facts seem only to confirm what we shall hereafter prove more directly, namely, that the menstrual hemorrhage is only a secondary phenomenon attendant on menstruation strictly so called, and that the capital phenomenon of this function consists in the periodical maturity and elimination of ova. There are some women in whom nothing else occurs besides this phenomenon and authentic cases are on record of women who have given birth to several children without having ever menstruated. Still stronger reasons are there for admitting the possibility of those cases in which, after the ovaries have for a length of time performed their monthly function unattended by any other phenomenon, menstrual hemorrhage at length occurs. Many other signs of puberty no less valuable than the flow of the menses serve to indicate the existence of that state when the menstrual flux is absent." (p. 89.)

As puberty approaches, the vesicles of De Graaf increase in number and size, and approach nearer to the surface of the ovary, the most superficial being usually the largest. M. Raciborski does not coincide in the opinion of M. Négrier, who describes these vesicles as presenting a plicated appearance at their edge, owing to their increase of size taking place more rapidly than that of the cell in which each is contained. The notion that each vesicle is contained in a distinct cell of the ovary, not simply imbedded in its stroma he conceives to be incorrect, and the only circumstances under which he has observed any wrinkling of the walls of the vesicle have been, when its contents have become partially absorbed; an occurrence which sometimes takes place in the course of protracted illness or amenorrhea, or at the time of the final cessation of the menses. When, under any of these circumstances, the contents of a vesicle become partially absorbed, a corresponding contraction of the external envelope of the ovary takes place, by which the parietes of the partially empty vesicle are compressed and thrown into wrinkles.

Long continued illness, it is well known, often has the effect of retarding the epoch of puberty, even after some of its precursory symptoms have appeared, or of suppressing the menstrual flux, although it may have already occurred once or twice. In such cases, the development of the Graafian follicles is found to have been arrested, as was observed by M. Raciborski in the case of two chlorotic girls who died between the ages of sixteen and seventeen; and in whom the Graafian vesicles, though as numerous as they should be at the period of puberty, were of extremely small size. In women in good health, however, menstruation when once established continues for the most part to recur regularly every month. Having noted the interval between the first two menstrual periods in eighty-seven women, M. Raciborski found that, in fifty-eight, this interval did not exceed one month. In two women the second menstruation followed six weeks after the first; in four, after an interval of two months; in five, after three months; in four, after four months; in one, at the end of five months; in another, at the end of eight months; in three, at the end of one year; and in another, after two years had elapsed. From these data it appears that, from the first appearance of menstruation, it recurs regularly every month in about two thirds of all healthy females.

It might be supposed, beforehand, that some relation would be found to exist between the length of the interval which separates the first two menstrual epochs, and the age at which the first appearance of the menses takes place. M. Raciborski's facts show that such a relation does actually exist, and that the interval between the first and second appearance of the menses is usually longer in those females who begin to menstruate at a comparatively late period, than in those in whom menstruation begins at an early age. As a further illustration of the connexion between the early occurrence of menstruation and the amount of uterine energy, it may be mentioned that,

"According to M. Pétrequin of Lyons, the tendency to irregularities of menstruation during the whole period of activity of the uterus bears a direct proportion to the lateness of its first appearance. This distinguished physician states that he has noticed irregularities of this kind only in one out of eight, of all females who began to menstruate at thirteen; while he has observed them in a fourth of those who began to menstruate at eighteen, in a third of those whose first menstruation did not occur till they were nineteen, and in three sevenths of those in whom the menses did not appear till the age of twenty." (p. 112.)

It is to be regretted that we do not know the numbers from which these conclusions are deduced.

The remainder of the first part of the work is occupied with remarks on the hygiene, and medical treatment of young females at the period of puberty; but as it contains little that has not been equally well said by others, we pass it over, and come to the second part, which treats of the various phenomena attending the final cessation of the menses. In this M. Raciborski distinguishes two separate elements: 1st. The cessation of the discharge of ova, and the consequent extinction of the reproductive faculty. 2d. The interruption to the long-acquired habit of periodical hemorrhage. The former he asserts—rather hastily, as we think—to be followed by results precisely the same as are produced by extirpation of the ovaries; to the latter he attributes all those ailments which are well known to attend in so many instances upon this period of female life. His remarks upon these subjects, however, are not of so much interest as his very elaborate description of the changes which the ovaries undergo at the period of the cessation of the menses. The progressive development of the ovaries and Graafian follicles characterizes, as we have already seen, the approach of puberty: their progressive atrophy marks the decline of the uterine functions. Many of the changes which the ovaries undergo at this time have been most accurately described by Roederer in his 'Icones Uteri;' and M. Raciborski has now rendered our acquaintance with them quite complete. It is a well-known fact that the ovaries of women who have ceased to menstruate undergo a marked diminution of their size, while their surface at the same time becomes extremely rough and irregular, so as closely to resemble the appearance of the kernel of a peach. Some observers, among whom is Mr. Girdwood, attribute this change to the ovaria having become as it were emptied of their contents by the successive maturation and discharge of ova during the long period of uterine activity; and in the rough, scarred, and corrugated appearance of the ovaries they see the result of their often-repeated cicatrization. M. Raciborski gives a different and, as we think, a more correct account of the process. He regards the corrugated appearance of the ovaries as the consequence of the atrophy of the Graafian follicles at the cessation of menstruation, and of the wrinkling of the elastic external envelope of the ovaries which necessarily follows. It will be seen, on examining ovaria which have thus begun to wither, that the fluid contents of the follicles have to a great extent become absorbed, so that they present the appearance of grayish or whitish sacs with wrinkled parietes. A slight traction with the forceps draws these sacs from the cavity in which they are contained, and leaves behind merely a circular excavation, lined by the external tunic of the Graafian follicle. When these changes are further advanced, the whole interior of the ovary is transformed into a cellulo-fibrous substance, presenting no trace of the natural structure of the gland, which is so much shrunken as scarcely to exceed the thickness of the broad ligament.

These transformations take place slowly, and the atrophy of the vesicles does not involve them all at once; but some which are in full activity will be seen close to others that have become greatly reduced in size. Thus do the results of anatomical investigation coincide with the daily-observed fact, that menstruation seldom ceases all at once, but grows irregular in its occurrence, and suffers pauses in its appearance before its final termination.

The remainder of the work is occupied with the exposition of the authors opinions and the details of his observations with reference to the periodical discharge of ova in the unimpregnated state, by the female of mammiferous animals. And here we come upon ground where M. Raciborski's honours are contested by another observer: by one of unquestionably higher scientific repute, and whose minutely-recorded observations, setting forth the steps by which he was led to the important discovery that he claims as entirely his own, certainly create a strong prejudice in his favour. M. Bischoff is unwilling to allow to M. Raciborski any higher merit than that which he shares in common with Dr. R. Lee, Mr. William Jones, Dr. Paterson, and MM. Négrier and Gendrin, of having proved the progressive development and final rupture of a Graafian vesicle at the time of menstruation and rut, and the subsequent formation of a *corpus luteum*, independent of impregnation. "The problem to be worked," as M. Bischoff remarks in his letter in the Gazette Médicale of Sept. 25, 1843, "was this: to prove that the escape of ova from the ovaries of mammifera and of the human female, does not depend on sexual intercourse, and on the influence of the semen, but on their own independent and periodical development. To prove this truth, it was necessary to follow the ova in different phases of development, to observe the rupture of the follicles of De Graaf, and to detect the ova in the tubes and oviducts in cases where sexual intercourse had not previously taken place. Now these direct proofs have been furnished by no one except myself. Neither M. Duverney, nor M. Pouchet, nor M. Raciborski, has traced the ova in the ovary and the oviduct." It is indeed unquestionably to M. Bischoff that the honour belongs, of having been the first to furnish that most satisfactory of all proofs, which is supplied by the discovery of the ovum during its transit through the Fallopian tube in an unimpregnated animal; though we cannot think him warranted in his assertion, that M. Raciborski had no notion of the whole truth when he published his first observations. The truth we believe to be, as is so often the case, between the two, for we by no means coincide with M. Raciborski in thinking that the changes which may be observed in the Graafian vesicles prove quite as much as can be gathered from discovering the ova external to the ovary. These changes may indeed, render extremely probable, but they cannot be regarded as proving that conception is merely the result of the accidental meeting of the semen with ova thus periodically discharged. That most important fact—the grand fact, indeed, to which the others are all subsidiary—could not be considered as actually demonstrated until observations had been made, such as those of Prof. Bischoff. Now *previous* to the publication of M. Bischoff's researches, it does not appear that M. Raciborski had ever detected ova external to the ovary; and we think, therefore, that the credit of having rendered certain a fact which previously was only in the highest degree probable, belongs to Professor Bischoff.

But, to return to the observations of M. Raciborski. It was in the sow that he first watched satisfactorily the progressive maturation and final rupture of the Graafian follicles. The peculiar structure of the ovary in that animal renders the alterations that the Graafian vesicles undergo remarkably evident. The first change seen to take place in them is the very considerable increase of those which lie nearest the surface of the ovary, when the animal is approaching that age at which she becomes capable of propagating. At the same time, the thickening of the investing membrane of the vesicles obscures their transparency, and their contents become more viscous and more abounding in granules. At the period of rut, the neighbourhood of some of these vesicles grows much congested, and the slight hemorrhage which often takes place into their cavity gives a bloody tinge to their contents. After continuing thus turgid for some days, the vesicles burst, the ovum escapes, they contract in size, and are found to contain only a little uncoagulated blood. The external tunic now contracts upon the empty cavity, and falling into folds, occasions the stellated appearance presented on making a section of a *corpus luteum*. Time greatly modifies the appearance of these bodies, and eventually they disappear; but during the whole period that the generative power continues, the ovaries will be found to contain Graafian follicles in different degrees of development: some which have long since been ruptured, and of which a single yellow tubercle is the only trace; others which, having burst more recently, present the radiated appearance with great distinctness; while others, nearer the surface, are growing turgid, and preparing for the discharge of their ova, when next the rutting season takes place; and some, still small and undeveloped, are imbedded in the interior of the ovary. Similar changes, though not quite so clearly marked, occur in the ovaries of other mammifera. In the case of a bitch, indeed, that had been shut up by herself, on manifesting the first symptoms of rut, and had been kept secluded from other dogs for a week before being killed, M. Raciborski not only found some follicles quite turgid, and one already ruptured, but likewise detected an ovum in each cornu of the uterus. The detail of this observation leads M. Raciborski into a lengthened exposition of his claims to the priority in this discovery, as opposed to those of M. Bischoff, on the merits of which we have already expressed our opinion. He next gives some interesting notices of analogous facts observed by other physiologists, but from which they had failed to draw all the inferences of which they would have admitted; and then quotes the ingenious and conclusive train of reasoning by which, though unsupported by direct experiment, M. Pouchet of Rouen, as early as 1835, had arrived at the conclusion, "that, beyond a doubt, the ovary, through the whole animal kingdom, discharges the ova independently of impregnation."

From the examination of the changes in the ovary of animals, M. Raciborski proceeds to investigate their alterations in the human female. In doing this he notices incidental observations by various writers, which, though not thoroughly understood by the persons who recorded them, may yet be appealed to at the present day as evidence in favour of the spontaneous rupture of the Graafian follicles. He next criticises the facts adduced by M. Gendrin, and also those by M. Négrier, as instances of the occurrence in the human subject, and shows the utterly inconclusive nature of the observations of the former writer. Even the far more correct account furnished by M.. Négrier has the great defect that the subject of his observations were not virgins; and consequently that the appearances he describes may have been the result of impregnation, and therefore cannot be regarded as establishing conclusively the fact of the spontaneous rupture of the Graafian follicle. In two instances M. Raciborski examined the ovaries of females in whom the hymen was perfectly intact, and who died just a month after they last menstruated. In each case one ovary was much larger than the other, and presented a Graafian follicle so turgid, and surrounded by so much congestion, as evidently to show that it would have speedily burst and given exit to the ovum. In no instance has M. Raciborski had the opportunity of examining the ovaries of any person who died while menstruating. M. Négrier, however, examined the ovaries of a young woman who died of violent scarlatina on the second day of the flow of her menses. No rupture of any Graafian follicle had yet taken place, though the coats of one that was very turgid were extremely attenuated. From this fact, as well as from the analogy afforded by animals in whom the rupture of the vesicles does not occur till after the state of heat has continued for some days, M. Raciborski concludes, with great probability, that the escape of the ovum in the human female does not take place till towards the end of the menstrual period. He further supports this opinion by reasons deduced from the appearance of the ovaries in women who have died a few days after menstruation. When we find him, however, asserting "that none but extremely slight traces of a corpus luteum are ever met with in women who have died in childbed," we cannot avoid concluding that his opportunities of examining the human ovary must have been but scanty; and we feel disposed to look with suspicion on arguments founded on such questionable data. M. Raciborski has collected evidence of another kind, however, in proof of the fact that the escape of the ova takes place towards the end of each menstrual period. Out of a large number of patients in the Clinique d'Accouchemens at Paris, whom he questioned as to the date of their last menstruation, and also of their having had sexual congress, fifteen only were able to furnish clear replies to both inquiries, but the answers of these fifteen all tend to substantiate M. Raciborski's opinion.

Such is an outline of M. Raciborski's observations; we will now turn to the carefully conducted investigations of Professor Bischoff. The various phenomena of generation had for years formed his especial study; but he long acceded undoubtingly to the old theory "which regards the escape of the ovum from the ovary, or the first condition essential to its development, as in some way or other necessarily connected with sexual congress." A repetition of the experiments of Nuck, Cruikshank, Haighton, Grassmeyer, Blundell, and Hausmann, first satisfied him that the escape of mature ova from the ovary was not determined by any action of the male semen. These experiments, of which he gives a minute detail, consisted in interrupting the communication between the vagina and ovaries by excision of part or the whole of the uterus, and then examining the bodies of the animals more or less speedily after they had had intercourse with the male. This first step of his investigation led him to the conclusion,

"That these experiments of my predecessors and myself prove most positively that, although the entrance of the male semen into the fallopian tube, and its access to the ovaries, and its consequent action be prevented, yet, on the occurrence of heat, (and in the above-mentioned cases after copulation,) exactly the same changes occur in the ova and ovaries as in the normal condition. The ova maturate, the Graafian vesicles become turgid and burst, and corpora lutea form, the ova escape and enter the oviduct, and some even of the phenomena of their development begin to take place. As the action, however, of the male semen on the ova is prevented, their development does not continue, but retrogrades, and the ova become dissolved and abortive. This last circumstance proves that the entire series of the phenomena is independent of impregnation, and has its origin in the natural course of development of the ova themselves. The influence of the aura seminalis, or the occurrence of resorption of the semen, or the existence of some more mystic agency in sexual congress, which some earlier observers have supposed to be proved by these experiments, would each be grounded on a supposition that I have shown to be untenable, from the fact of the semen coming into actual contact with the ova; and in these cases, too, is especially disproved by the circumstance that these ova, with which such actual contact of the semen was prevented, were insusceptible of development, and perished. Had the influence of the semen and sexual intercourse been the cause of the escape of the ova, and of the development of the *corpora lutea* in these cases, the ova, also, must have been impregnated, and their development must have gone on further. I think, therefore, that notwithstanding the occurrence of sexual intercourse, the independent development of the ova is proved by these experiments." (pp. 17-18.)

It may be doubted whether Professor Bischoff has not drawn a wider inference from these experiments than they quite warrant; since it is perfectly conceivable that the venereal orgasm might exert such an action on the whole system as to occasion the detachment of an ovum, though from the obliteration of the fallopian tube, the access of the male semen, and its consequent impregnation be impossible. The following experiment, however, satisfactorily proves the correctness of this opinion. A young and healthy bitch that had never pupped, was watched for several days after coming into heat, and then was lined. Immediately after coitus, Professor Bischoff extirpated the left cornu of the uterus, together with the ovary and Fallopian tube of the same side. He now made a careful microscopic examination of the parts he had removed, and found that semen had reached as far as the upper angle of the cornu of the uterus, and that the spermatozoa were moving. There was no trace of semen in the tube, but the ovary already presented burst Graafian follicles and distinct corpora lutea. Five ova were discovered in the Fallopian tube, along which they had advanced about two inches.

After the lapse of twenty hours the bitch was killed, Professor Bischoff having calculated that time had then passed sufficient for the semen to reach the remaining ovary, when five small openings were observed in the right ovary, and five corpora lutea were contained within it; five ova too were contained in the Fallopian tube, and had reached the middle of its canal. Spermatozoa, still in a state of activity, were found to have penetrated about three lines beyond the uterine orifice of the tube, but none were found in the rest of the tube, nor were there any about the ova, which therefore, without doubt, were not yet impregnated.

It would seem, from this observation, that the lapse of time between the escape of the ova and their fructification varies: since in other instances six, eighteen, or twenty hours after coition, the Graafian vesicles were found still closed, and the semen was discovered to have traversed the whole length of the Fallopian tube, and to have reached the ovary. For this difference there would seem to be no other assignable cause, than the individual peculiarities of different animals, some of which admit the approaches of the male at an earlier period of rut than others: some while the ova are still contained in the ovary, others not until they have already entered the Fallopian tube. It is not possible to fix definitely the period during which the impregnation of the ova is possible. "But since bitches generally admit the approaches of the male during eight days, and since the first distinct indication of the development of the ova, namely the cleavage of the yelk, begins in the lowest part of the Fallopian tube, at which they arrive about the seventh or eighth day, this would seem to fix the limits during which impregnation is possible in the dog." (p. 22.) From this too it is evident, how thoroughly uncertain a mode of calculating the date of the escape of ova from the ovary, is the hitherto customary one of reckoning from the first sexual congress, and that it can at the most present only an approximation to the real results.

Still more conclusive, however, are those experiments in which animals were killed during the state of heat, but without having been allowed access to the male, and in which nevertheless Graafian vesicles were found ruptured, and corpora lutea developed; and the ova themselves were detected at some point or other in the Fallopian tubes. Some observations of Hausmann indicate this result, but the strange prejudice that led him to deny the existence of the ovarian ovum of course diminishes their value, and confines the evidence afforded by them to the two former points,—the rupture of Graafian vesicles, and the development of corpora lutea. Professor Bischoff relates the particulars of the observations which he made with the view of ascertaining this point. The first was made on a lamb which had never before been in rut, and which was killed some hours after it entered into that state. A Graafian vesicle was found ruptured in the right ovary, and the ovum was detected in the corresponding Fallopian tube. The second experiment was made on a bitch which was watched while in heat, until she appeared willing to admit the male, but was then prevented from so doing. Two days afterwards Professor Bischoff extirpated the left ovary and Fallopian tube, and closed the wound by suture. No Graafian vesicle had burst, but four were extremely turgid, and from 2 to 2½ Paris lines in diameter.

"I dissected them carefully," says the author, "from the stroma of the ovary, and then laid them upon a plate of glass. As I opened the first, an ovulum, still surrounded by its disc, and 0·0078 of a Paris inch, or 11-50th of a millimeter, in diameter escaped with the fluid. To my surprise, however, this Graafian vesicle contained a second ovulum with its disc, measuring 0·0081 of a Paris inch, or 1-4th of a millimeter, in diameter. The three other follicles each contained an ovum of about the same size. The inner surface of the Graafian follicle was already beset with delicate granulations, the first traces of the mass that forms the corpus luteum, which, as it seems to me, develops itself from the cells of the membrana granulosa. I observed here, very distinctly, in several Graafian vesicles, the manner in which the ova are contained within them. The cells of the disc form a small cone, whose rounded apex receives the ovulum with which it projects free into the fluid, filling the cavity of the Graafian follicle: while it is attached by its base to the wall of the follicle, probably just in that situation where its rupture subsequently takes place.

"These ova, however, did not appear to be perfectly mature and ready to escape. For not only were the walls of the Graafian vesicles not very much attenuated, but the cells of the disc were not yet elongated into those fibrils, the existence of which betokens the full maturity of the ovum. The ova, when entirely detached by means of the needle from the *zona*, measured 0·0060, or 0·0065 of a Paris inch, or from 8-50th to 1-6th of a millimeter, in diameter. In all of these the yelk completely filled the interior of the *zona*, except at one place, where, for a very small extent, the yelk-granules had retreated, as though that were the situation of the germinal vesicle. I was nevertheless unable to distinguish it, either while the ova were still closed, or when I had opened them under the compressorium. I feel therefore quite certain that had the bitch been lined at this time, the spermatozoa would have had time to reach as far as the ovaries before the Graafian vesicles would have ruptured." (p. 29-30.)

Five days afterwards the bitch was killed. Four large corpora lutea were found in the ovary, and on careful examination the four ova were discovered in the Fallopian tube, and already three inches distant from its abdominal orifice. The ova still presented the disc surrounding the *zona*, but its cells no longer retained their natural appearance, and their solution had evidently commenced.

"The ova had somewhat increased in size, being 0·0090 to 0·0097 of a Paris inch in diameter; but the yelk appeared somewhat shrunken, and no longer filled up the *zona*. It retained moreover its usual form, and not a trace of cleavage was evident in it. In one ovum, (that which was nearest to the commencement of the Fallopian tube,) there was evident in the interspace between the yelk and the *zona*, that large vesicle or granule measuring 1-140th of a Paris line, or 1-62d of a millimetre, which I have already pointed out as being the nucleus of the germinal vesicle. In the three other ova, nothing of the kind was observable. I saw no trace of the germinal vesicle in any of the ova." (p. 31.)

We certainly agree with Professor Bischoff in the opinion that it can hardly be possible to display more clearly than has been done by this twofold observation made on the same animal the whole process of the maturation and escape of ova during rut, and its independence of coition. Still further proof, however, is afforded by the examination of the genitals of the sow, of which three instances are recorded by Professor Bischoff. The existence of the same law through the whole class of mammifera is further illustrated by an observation on the rat, detailed, as all his other observations are, with a minuteness and precision wholly unlike the general terms in which M. Raciborski's observations are couched, and which leave the reader at a loss as to the number and nature of the facts on which his assertions are grounded.

"From all that has been said, it is quite certain that during rut, even when coition does not take place, the ova of mammifera become detached from the ovary, and enter the Fallopian tube where they perish; but corpora lutea form exactly as when sexual intercourse and impregnation have occurred." (p. 38.)

To the latter clause of this sentence indeed, we still cannot avoid withholding our assent; the identity of the corpora lutea formed independently of impregnation with those which succeed to fruitful intercourse between the sexes, appears to us, to say the least, not yet proven. The recent observations of Dr. Ritchie indeed seem to indicate that the whole subject of the structure and varieties of the corpus luteum requires a new and most careful investigation.

We have now noticed the whole of the evidence in support of this new theory of generation afforded by the investigations of Professor Bischoff. The remainder of his tract is occupied with pointing out the probability that the same phenomena which have been proved to take place in animals, do likewise occur in the human subject, and that menstruation in the human female is but a slightly modified expression of the same occurrences as take place in animals during the state of rut. We cannot, however, enter now upon the detail of these arguments, for the mere relation of Professor Bischoff's observations has already led us further than we intended.

For the present then we take leave of the subject, though soon to return to it; since almost every day some new light is shed on its mysteries; or at least inquiries which seem to produce no other fruit, show how much of what has appeared to be most plausible, or most clearly proved, rests either on groundless assumption, or on investigations vitiated by some preconceived theory, or leading to results capable of various explanation.

